# Vascular Protection by Angiotensin Receptor Antagonism Involves Differential VEGF Expression in Both Hemispheres after Experimental Stroke

**DOI:** 10.1371/journal.pone.0024551

**Published:** 2011-09-01

**Authors:** Weihua Guan, Payaningal R. Somanath, Anna Kozak, Anna Goc, Azza B. El-Remessy, Adviye Ergul, Maribeth H. Johnson, Ahmed Alhusban, Sahar Soliman, Susan C. Fagan

**Affiliations:** 1 Program in Clinical and Experimental Therapeutics, University of Georgia College of Pharmacy, Augusta, Georgia, United States of America; 2 Charlie Norwood VA Medical Center, Augusta, Georgia, United States of America; 3 Departments of Physiology, Georgia Health Sciences University, Augusta, Georgia, United States of America; 4 Department of Neurology, Georgia Health Sciences University, Augusta, Georgia, United States of America; 5 Department of Biostatistics, Georgia Health Sciences University, Augusta, Georgia, United States of America; Northwestern University, United States of America

## Abstract

We identified that the angiotensin receptor antagonist, candesartan, has profound neurovascular protective properties when administered after ischemic stroke and was associated with a proangiogenic state at least partly explained by vascular endothelial growth factor A (VEGFA). However, the spatial distribution of vascular endothelial growth factor (VEGF) isoforms and their receptors remained unknown. Protein analysis identified a significant increase in vascular endothelial grow factor B (VEGFB) in the cerebrospinal fluid (CSF) and the ischemic hemispheres (with increased VEGF receptor 1 activation) of treated animals (p<0.05) which was co-occurring with an increase in protein kinase B (Akt) phosphorylation (p<0.05). An increase in VEGFA protein in the contralesional hemisphere corresponded to a significant increase in vascular density at seven days (p<0.01) after stroke onset. Vascular restoration by candesartan after stroke maybe related to differential regional upregulation of VEGFB and VEGFA, promoting a “prosurvival state” in the ischemic hemisphere and angiogenesis in the contralesional side, respectively. These vascular changes in both hemispheres after effective treatment are likely to contribute to enhanced recovery after stroke.

## Introduction

Reperfusion therapy with either fibrinolysis or mechanical clot removal is the current standard of care for acute treatment of ischemic stroke [1]. However, the treatment is limited by a short time window and a fear of reperfusion injury, including hemorrhage development. Vascular protection (reducing hemorrhage and edema formation) has emerged as a promising strategy to improve outcome and hasten recovery from acute ischemic stroke. Many potential targets have been proposed [2] and growth factors, in particular VEGF, have been identified as both neuroprotective and vascular protective [3]. However, VEGF has the undesirable effect of increasing vascular permeability, leading to increased edema and hemorrhage in some models [4]. In our previous work, we demonstrated profound neurovascular protection with candesartan, an angiotensin receptor antagonist, and this was associated with an increase in VEGF expression (as detected by a nonspecific enzyme-linked immune sorbent assay (ELISA)) in the ischemic hemisphere but decreased vascular permeability. The CSF from candesartan treated animals stimulated tube formation in brain endothelial cells but this proangiogenic effect was only partly blocked by a VEGFA blocking antibody [5]. We used a rat model to determine the role of VEGF isoforms and their receptors in vascular protection after experimental stroke.

## Results

### Quantitative PCR array

**Figure 1:**
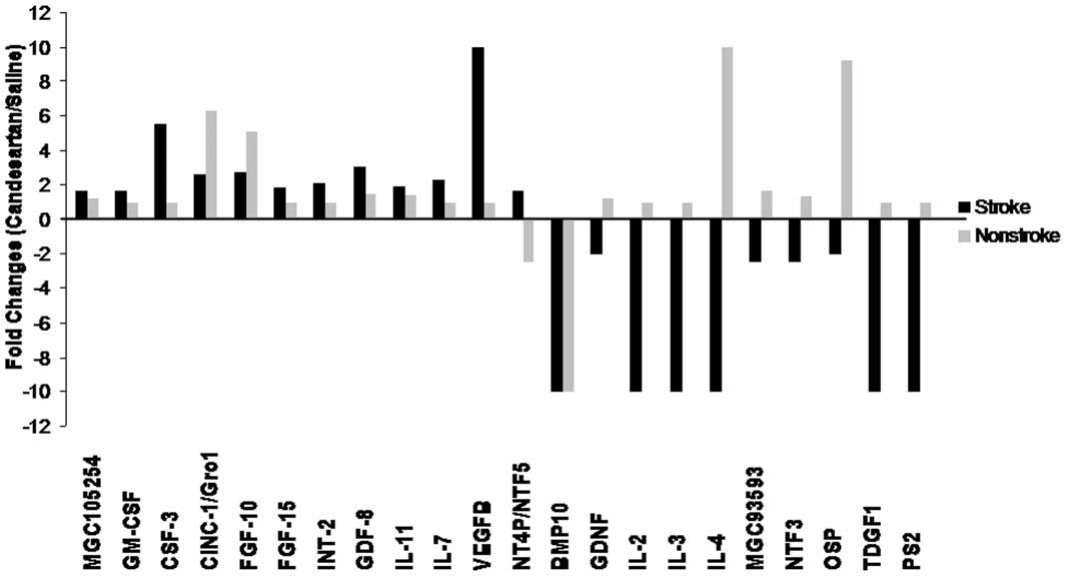
PCR array. PCR array was performed on the brain tissue homogenates from candesartan and saline treated animals (n = 2). Brain tissue samples were taken from both hemispheres as shown in Figure 4a. Regulated genes are presented as fold changes. Positive values mean upregulated and negative values mean downregulated.12 genes were upregulated and 10 genes were downregulated more than 1.5 fold in candesartan-treated animals, compared to saline-treated animals. VEGFB was particularly upregulated by candesartan.

Of the total of 84 genes tested in the PCR array, 12 reached the threshold for upregulation and 10 reached the threshold for downregulation more than 1.5 fold by candesartan when compared to the saline-treated animals(n = 2). The largest increase was in VEGFB (Figure 1). These data suggest that candesartan enhances the expression of VEGFB mRNA in a PCR array.

### Hemorrhage

**Figure 2:**
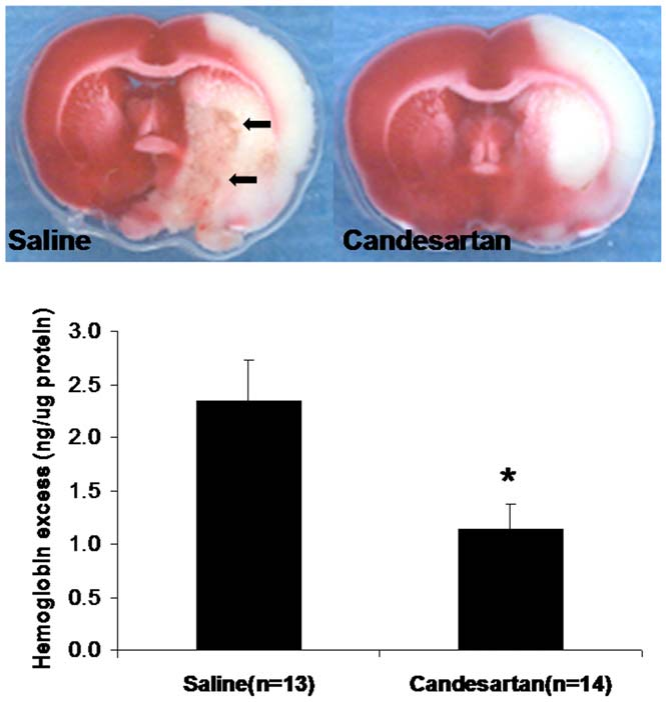
Hemorrhage. ELISA was performed on the brain tissue form candesartan and saline treatment animals 24 hours after stroke onset. Treatment with candesartan significantly decreased hemoglobin excess (*P<0.05) in the ischemic brain, as compared with the saline-treated animals. Arrows indicate hemorrhagic transformation of the ischemic lesion in a representative animal.

Candesartan treatment significantly lowered hemoglobin excess (bleeding) in the ischemic hemisphere by about 50% (P = 0.013) (Figure 2) compared with saline-treated animals.

### Vascular Endothelial Growth Factor Expression in CSF

**Figure 3:**
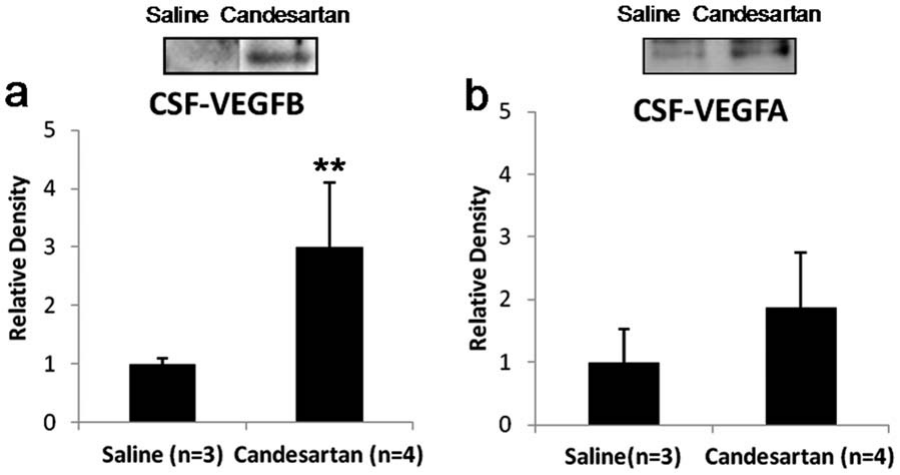
VEGF expression in CSF. Western blot was performed on the cerebrospinal fluid (CSF) from candesartan and saline treatment animals at 24 hours after stroke onset. Treatment with candesartan significantly increased VEGFB (Figure 3a, **P<0.01) protein expression in the CSF. However, no significant difference was found for the VEGFA protein expression (Figure 3b).

Since our earlier work suggested a proangiogenic effect in the CSF [5], we compared the concentrations of VEGFA and VEGFB in the CSF of both candesartan and saline treated animals at 24 hours after stroke. Approximately 3 fold higher VEGFB protein was evident (P = 0.007) (Figure 3). These data suggest that candesartan increases VEGFB protein in the CSF 24 hours after experimental stroke. The difference in VEGFA was not significant (Figure 3).

### Vascular Endothelial Growth Factor Expression in the Brain

**Figure 4:**
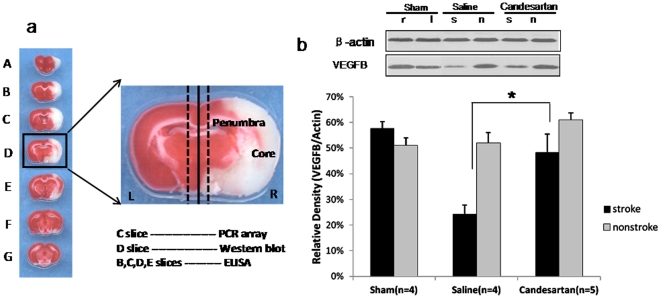
VEGFB expression in the brain. Western blot was performed on brain tissue from sham animals, candesartan and saline treated animals at 24 hours after stroke onset. Brain tissue samples were taken as shown in Figure 4a.There was a significant interaction between treatment groups. For the saline-treated animals, the ischemic hemisphere had lower VEGFB expression compared with the nonischemic hemisphere (P = 0.012).However, there was no significant difference between the two hemispheres for the sham animals (P = 0.88) or candesartan-treated animals (P = 0.29). Candesartan treatment significantly preserved VEGFB protein expression in the ischemic hemisphere (Figure 4b,*P<0.05) as compared with saline-treated animals.

**Figure 5:**
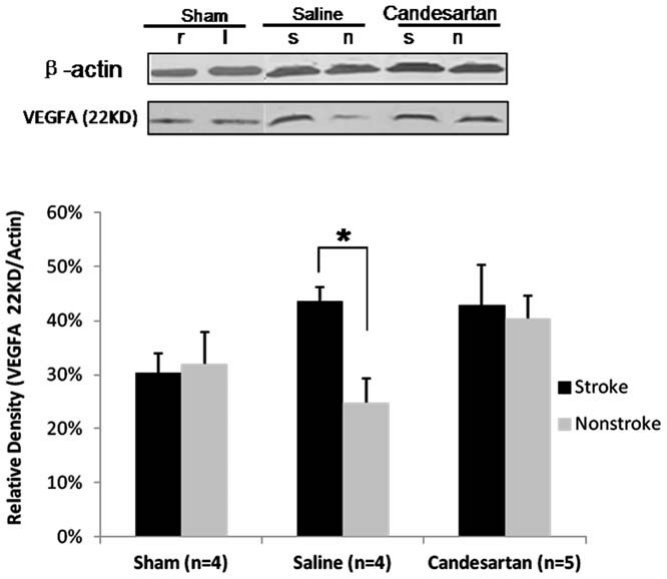
VEGFA expression in the brain. The ischemic hemisphere had higher VEGFA expression compared with the nonischemic hemisphere (Figure 5, *P<0.05) in the saline-treated animals. However, there was no significant difference between the two hemispheres in the sham animals (P = 0.78) or candesartan-treated animals (P = 0.63). Moreover, the interaction between treatment groups and brain hemisphere (P = 0.06) almost reached statistical significance. Treatment with candesartan showed a statistical trend of increasing VEGFA protein expression in the nonischemic hemisphere (Figure 5, P = 0.062).

We also compared VEGF expression in the brain tissue in the sham animals and saline or candesartan-treated animals at 24 hours after stroke. For the VEGFB expression, there was a significant interaction between treatment groups and brain hemisphere (P = 0.010). For the saline treated animals, the ischemic hemisphere had lower VEGFB protein compared with the nonischemic hemisphere (P = 0.012) (Figure 4b). However, there was no significant difference in VEGFB expression between the two hemispheres in the sham animals (P = 0.88) or candesartan-treated animals (P = 0.29). Treatment with candesartan resulted in a significant preservation of VEGFB in the ischemic hemisphere (P = 0.010) (Figure 4b), such that it approached that of the nonischemic side. For VEGFA, the interaction between treatment groups and brain hemisphere (P = 0.06) almost reached statistical significance. The ischemic hemisphere had higher expression compared with that of the nonischemic hemisphere in the saline group (P = 0.007) (Figure 5). There was no significant difference between the two hemispheres for the sham (P = 0.78) or candesartan groups (P = 0.63). Candesartan treatment showed a statistical trend of increasing VEGFA protein expression in the nonischemic hemisphere (Figure 5). These data suggest that candesartan preserves VEGFB protein in the ischemic hemisphere and increases VEGFA protein in the nonischemic hemisphere.

### Vascular Endothelial Growth Factor Receptor Expression in the Brain

**Figure 6:**
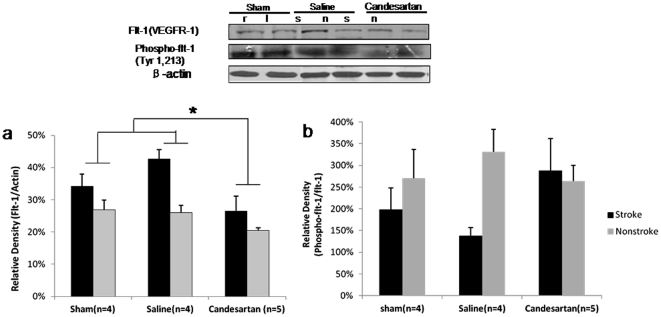
VEGFR-1 expression in the brain. Treatment with candesartan significantly decreased VEGFR1(flt-1) expression in both hemispheres compared with saline-treated or sham animals (Figure 6a, *P<0.05). VEGFR1 activation, as determined by their phosphorylation status, was not significantly different among sham animals, candesartan, and saline-treated animals (Figure 6b). Candesartan treatment appeared to visually increase VEGFR1 activation in the stroke side, but this did not achieve significance (Figure 6b).

**Figure 7:**
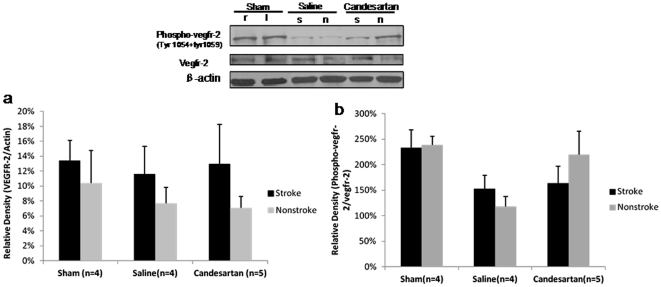
VEGF-2 expression in the brain. Candesartan treatment did not result in any changes in VEGFR2 protein expression (Figure 7a) or VEGFR-2(flk-1) activation (Figure 7b), as determined by the phosphorylation status, compared with saline-treated or sham animals in both hemispheres (Figure 7b).

VEGF isoforms interact with VEGFR1 and VEGFR2 differently. VEGFB exerts is prosurvival effects predominantly through VEGFR1 and VEGFA induces angiogenesis primarily through VEGFR2 [6]. The degree of phosphorylation of these receptors is used to quantify their activation. VEGFR1 (flt-1) was significantly higher in the stroke hemisphere when compared to the nonstroke side (P = 0.0036) for all treatment groups (Figure 6a). Candesartan resulted in a significant decrease in VEGFR1 in both hemispheres (P = 0.014 for treatment effect) (Figure 6a) compared to the sham or saline groups (P = 0.040 and P = 0.005, respectively) (Figure 6a). Candesartan treatment did not result in any changes in VEGFR2 (flk-1) expression in either hemisphere (Figure 7a).

Candesartan treated animals showed a visual increase in phospho-flt-1 (activation of VEGFR1) expression in the stroke hemisphere (Figure 6b) and increased phospho-flk-1(activation of VEGFR 2) expression in the nonstroke hemisphere (Figure 7b), but neither achieved statistical significance. However, it is likely that the number of animals included were too few to give the study enough statistical power to demonstrate any differences, if they exist (Figure 6b,7b).

These data suggest that candesartan may up-regulate VEGFR1 activation in the ischemic hemisphere and VEGFR2 activation in the nonischemic hemisphere. This corresponds well to the increased activity of the prosurvival VEGFB in the area of the ischemia and the more proangiogenic VEGFA on the contralateral side.

### Akt and p38 MAPK Pathway Assay

**Figure 8:**
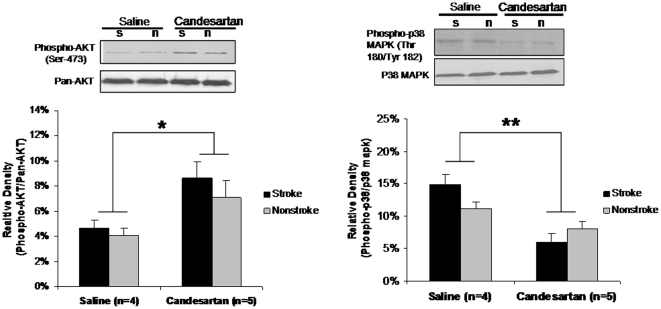
Akt and P38 pathway assay. Candesartan treatment significantly increased protein kinase B (AKT) activation (Figure 8a, *P<0.05) and inhibited P-38 kinase activation (Figure 8b, **P<0.01) in both hemispheres.

Candesartan treated animals had an increased activation of the prosurvival protein kinase B (Akt) (P = 0.034) (Figure 8a) and a decreased activation of the apoptotic p38 kinase (P = 0.0003) (Figure 8b), as determined by their phosphorylation status, in both hemispheres. These data suggest that the upregulation of both VEGFA and VEGFB resulted in a prosurvival state.

### Vascular Density

**Figure 9:**
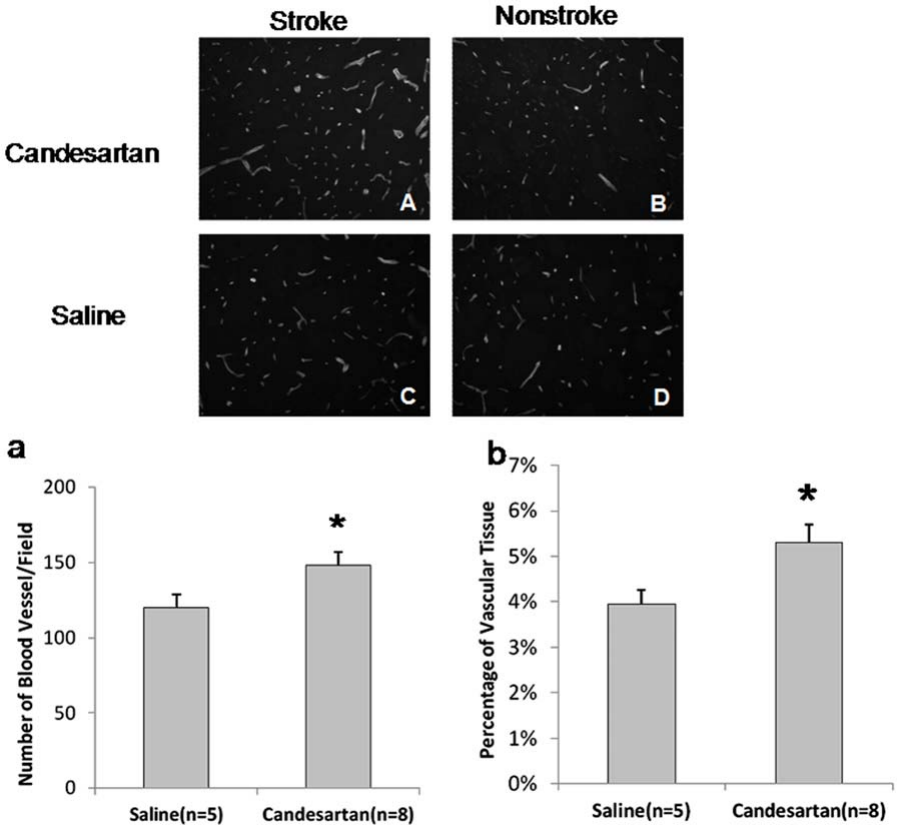
Vascular density. Immunohistochemical staining was performed on the brain tissue from candesartan and saline treated animals 7 days after stroke onset. Treatment with candesartan significantly increased the number of blood vessels (*P<0.05) (Figure 9a) and the percentage of vascular tissue (*P<0.05) (Figure 9b) compared with saline-treated animals in the nonischemic hemisphere.

Candesartan treated animals had a significantly higher percentage of vascular tissue (P = 0.020) (Figure 9b) and number of blood vessels (P = 0.045) (Figure 9a) compared with saline treated animals in the nonischemic hemisphere. We have already published the data from the ischemic hemisphere [5]. These data suggest that candesartan increases vascular density in both hemispheres after stroke.

## Discussion

Functional magnetic resonance imaging (FMRI) in human stroke victims clearly demonstrates the involvement of both ipsilateral and contralateral hemispheres in recovery after stroke [7].This study reports, for the first time, that candesartan promotes the differential expression of both VEGFA and VEGFB and their receptors in the brain and may be responsible for the creation of a prosurvival state in both hemispheres after stroke. Our initial finding of a proangiogenic factor in the CSF, only partly blocked by a VEGFA antibody [5], led us to studies aimed at determining the impact of these findings on both hemispheres after stroke. With isoform-specific protein analysis on brain homogenates and CSF, we were able to confirm a predominant induction of VEGFB in the ischemic hemisphere with a concurrent increase in VEGFA in the nonischemic hemisphere. Phosphorylation of the relevant VEGF receptors in both the ischemic and contralesional hemispheres supports this finding. This combination may be linked to recovery by enhancing function in both hemispheres. Our earlier report of increased VEGF in the ischemic hemisphere of candesartan treated animals [5], reflected the inability of the nonspecific ELISA assay to differentiate between VEGFA and VEGFB and is a more common way to report VEGF quantification in the stroke literature. The results of the PCR array data reported here identified VEGFB as an important contributor to this finding in the ischemic hemisphere. We detected no hint of increased gene expression of VEGFA after stroke and candesartan, however. This may have been due to the fact that we were focused on the changes in the ischemic hemisphere.

Although an understudied cousin of VEGFA, VEGFB has already been shown to be important in recovery after ischemic stroke [8] and in other models of neuronal and vascular injury [9].It has been shown to be antiapoptotic [10],[11]and is only mildly angiogenic [11]. It has the protective effects of VEGFA [3] without the increase in vascular permeability that can be devastating in acute stroke [12],[13].

We are first to report a significant increase in protein kinase B (Akt) activation with angiotensin antagonism after stroke, with a reciprocal decrease in p38 MAP kinase. A reciprocal regulation of cell survival kinase Akt and stress induced p38 MAP kinase has been shown to be necessary for the creation of a prosurvival state. Our findings are supported by others who have reported a favorable effect of candesartan on Akt phosphorylation in vascular smooth muscle cells [14]. The prosurvival state is regulated via Akt-mediated phosphorylation and inhibition of apoptosis signal regulating kinase1 (ASK1), an upstream activator of p38 MAP kinase [15]. It has also been shown that p38 MAP kinase can act as a “molecular switch”, such that inhibition results in angiogenesis but blocked hyperpermeability [16]. This may offer an additional explanation why the increase in VEGF and a proangiogenic state we reported after stroke and candesartan was accompanied by an actual decrease in permeability [5].

Angiogenesis in the nonischemic brain after stroke, as reported here in treated animals, may be a key component of functional recovery. Whether this is caused by, or related to, the increase in VEGFA and VEGFB is under investigation. It is possible that this “prosurvival state”, demonstrated in the candesartan-treated animals and appearing to be very similar to that caused by ischemic postconditioning [17], is the mediator of the functional recovery. The differential regional induction of the isoforms of VEGF may be neuroprotective, neurorestorative, or only important in vascular protection. This will have to be determined through extensive genetic and pharmacologic studies both in vitro and in vivo.

In our study, we only focused on the effects of VEGFA,VEGFB and their corresponding receptors to stroke recovery. However, there is a possibility that other VEGF isoforms and VEGF receptors might contribute to the recovery [18],[19]. Long-term studies can better address the relationship between VEGF expression, vascular restoration and functional outcome after stroke. These studies are ongoing where an experimental stroke model with a smaller infarct size will be utilized to enhance the ability of the animals to thrive for prolonged periods after injury.

In summary, our data indicate that VEGFB and VEGFA and their receptors are up-regulated by candesartan in the brain after ischemia and the changes are seen differentially in both hemispheres. Inhibition of p38 MAP kinase allows the positive benefits (vascular protection and angiogenesis) of the VEGF isoforms to occur without accompanying hyperpermeability. Harnessing the restorative properties of the vasculature in both hemispheres is a promising therapeutic strategy for ischemic stroke patients.

## Methods

The experimental protocol was approved by the institutional Animal Care and Use Committee of the Charlie Norwood Veterans Affairs Medical Center (09-04-008). Sixty adult male Wistar rats (Charles River Breeding Company, Wilmington, Mass), weighing between 270 and 300 g, were divided into sham, saline and candesartan treatment groups. Temporary (3-hour) middle cerebral artery occlusion (MCAO) was achieved using the intraluminal suture model [20] under isoflurane anesthesia. 19–21 mm silicon coated suture (403756PK10, Doccol Corporation, Redlands, CA) was introduced from the external carotid artery (ECA) lumen into the internal carotid artery (ICA) to block the origin of the right middle cerebral artery (MCA). The animals were kept under anesthesia for only 15 minutes for the surgical procedure. Temperature was maintained at 37±0.5°C using a controlled heating system. At reperfusion, a single dose of 1 mg/kg candesartan(Astra-Zeneca) or saline control was given intravenously through a tail vein at a volume of 1 mL/kg. The candesartan dose was previously shown to be neurovascular protective [5]. After middle cerebral artery occlusion (MCAO), animals were divided into four sets. The first set of animals were sacrificed at 24 hours after stroke and brain tissue sliced at 2 mm intervals (B–E), then separated into two hemispheres and snap frozen and stored at minus 80 degree for quantitative PCR array and western blot studies (n = 13). A second set of animals had CSF collected from the cisterna magna (n = 7) at 24 hours. The third set of animals had brain tissue collected for quantification of hemoglobin (n = 27) at 24 hours and the last set of animals had brain tissue collected for immunohistochemical analyses (n = 13) at 7 days after stroke.

### Hemorrhage Determination

At 24 hours after the onset of MCAO, animals were anesthetized with a cocktail of ketamine (45 mg/kg) and xylazine (15 mg/kg) via intramuscular injection then perfused with saline, sacrificed and their brains were removed. The brain tissue was sliced into seven 2 mm-thick slices (A–G) in the coronal plane. The ischemic and non-ischemic hemispheres of the slices for ELISA were separated and processed, using the non-ischemic side as a control. After homogenizing the slices in the core of the infarct (B–E) and taking the supernatants, ELISA was performed to measure the hemoglobin in the brain tissue [21].

### Quantitative PCR array analysis

Frozen brain tissue samples (C slice) from both hemispheres (Figure 4a) were homogenized in TRIzol reagent (Invitrogen Life Technologies, Carlsbad, CA). Total RNA was isolated using the TRIzol reagent (Invitrogen) followed by RNeasy Mini Kit (QIAGEN) purification according to the manufacturer's instructions. Total RNA was subjected to cDNA preparations using RT2 first strand kit. Gene assay was performed using the Rat Growth Factors RT2 Profiler™ PCR Array kit (SABiosciences). Of 84 genes related to growth factors, a threshold of 1.5 was used to identify genes of interest.

### Western blot analysis

Frozen brain tissue samples (D slice) from both hemispheres (Figure 4a) were homogenized in lysis buffer (RIPA). Homogenates (or CSF samples) were centrifuged, protein concentration determined, and 100 µg of protein per lane was subjected to SDS-PAGE and transferred to nitrocellulose membranes. Membranes were then blocked at room temperature for 1 h in 5% bovine serum albumin (BSA) and incubated with rabbit polyclonal anti-VEGFB (ab58461,abcam, 1∶250, Cambridge, MA), VEGFA (07-1376, Millipore, 1∶200, Billerica, MA), rabbit antiphospho-S473Akt (9271; Cell Signaling,1∶500, Danvers, MA), polyclonal rabbit antipan Akt (9272; Cell Signaling,1∶1000, Danvers, MA), phospho-p38 MAPK Kinase (Thr180/Tyr182) antibody (9211;Cell Signaling,1∶500, Danvers, MA), or p38 MAPK antibody (9212;Cell signaling,1∶1000, Danvers, MA), rabbit polyclonal VEGF receptor 1 (VEGFR1) antibody (ab2350;abcam,1: 100, Cambridge, MA), anti-phospho-Flt (Tyr 1213) (07-758;Millipore; 1;750, Billerica, MA), rabbit polyclonal VEGF receptor 2 (VEGFR2) antibody (ab39256; abcam,1: 750, Cambridge, MA) and anti-phospho-VEGF receptor 2 (Tyr 1054+Tyr 1059) antibody (ab5473; abcam,1∶1000, Cambridge, MA). Each was diluted in 0.1% Tween 20/20 mM Tris-buffered saline (TBS) or 5% BSA. Protein loading was controlled with rabbit anti-β actin (A5060; Sigma,1∶5000, St. Louis, MO). Protein levels were analyzed densitometrically, using Image J software and were normalized to loading controls.

### Vascular Density Determination

Seven days after MCAO and treatment with either candesartan or saline, rats were anesthetized with an 85% ketamine/15% xylazine combination and transcardially perfused with normal saline followed by 4% paraformaldehyde. Brains were quickly removed and fixed in 4% paraformaldehyde for 3 hours and then sliced into 2-mm coronal sections. These sections were further fixed in 4% paraformaldehyde for 24 hours and then transferred to 70% isopropyl alcohol. The immunohistochemical analyses using a primary antibody for laminin (rabbit polyclonal; Novus Biologics, Littleton, Co), a biotinylated secondary antibody (Vector Labs #B1205), and fluorescence detection, were performed on slide-mounted, paraffin-embedded 5 µm thick sections taken as previously described [5]. In the striatum, both the number of individual blood vessels and the vascular density of these vessels were determined with Image J (US National Institutes of Health, Bethesda) image processing program. Three images per area per animal were analyzed and vascular density and the number of vascular profiles averaged.

### Statistical analysis

All data are presented as mean ± standard error (SEM). Data were evaluated for normality and data transformation or nonparametric analysis approach was considered if data were not normal. A 3 X 2 mixed model repeated measures analysis of variance (RMANOVA) was used to study the effect of treatment (sham, saline, and candesartan) and brain hemisphere (ischemic vs. nonischemic) and their interaction on VEGFA, VEGFB, VEGFR1, and VEGFR2. A 2 X 2 RMANOVA was used to analyze the effect of treatment (saline vs candesartan) and brain hemisphere (ischemic vs nonischemic) on AKT and P38. A Tukey's adjustment for multiple comparisons was used when interpreting significant interaction effects. We also investigated the candesartan effect on hemorrhage in the brain, VEGF expression in the CSF, and vascular density compared with saline control using two-sample *t*-test. Statistical significance was determined at p<0.05 for all analyses. All analyses were performed using SAS 9.2 (SAS Institute Inc., Cary, NC).
